# Associations Between Added Sugar Intake and Risk of Four Different Cardiovascular Diseases in a Swedish Population-Based Prospective Cohort Study

**DOI:** 10.3389/fnut.2020.603653

**Published:** 2020-12-23

**Authors:** Suzanne Janzi, Stina Ramne, Esther González-Padilla, Linda Johnson, Emily Sonestedt

**Affiliations:** ^1^Nutritional Epidemiology, Department of Clinical Sciences Malmö, Lund University, Lund, Sweden; ^2^Cardiovascular Research, Department of Clinical Sciences Malmö, Lund University, Lund, Sweden

**Keywords:** cardiovascular diseases, added sugar intake, dietary sugar, sugar-sweetened beverages, cardiometabolic risk factors

## Abstract

**Aims:** Although diet is one of the main modifiable risk factors of cardiovascular disease, few studies have investigated the association between added sugar intake and cardiovascular disease risk. This study aims to investigate the associations between intake of total added sugar, different sugar-sweetened foods and beverages, and the risks of stroke, coronary events, atrial fibrillation and aortic stenosis.

**Methods:** The study population consists of 25,877 individuals from the Malmö Diet and Cancer Study, a Swedish population-based prospective cohort. Dietary data were collected using a modified diet history method. National registers were used for outcome ascertainment.

**Results:** During the mean follow-up of 19.5 years, there were 2,580 stroke cases, 2,840 coronary events, 4,241 atrial fibrillation cases, and 669 aortic stenosis cases. Added sugar intakes above 20 energy percentage were associated with increased risk of coronary events compared to the lowest intake category (HR: 1.39; 95% CI: 1.09–1.78), and increased stroke risk compared to intakes between 7.5 and 10 energy percentage (HR: 1.31; 95% CI: 1.03 and 1.66). Subjects in the lowest intake group for added sugar had the highest risk of atrial fibrillation and aortic stenosis. More than 8 servings/week of sugar-sweetened beverages were associated with increased stroke risk, while ≤2 servings/week of treats were associated with the highest risks of stroke, coronary events and atrial fibrillation.

**Conclusion:** The results indicate that the associations between different added sugar sources and cardiovascular diseases vary. These findings emphasize the complexity of the studied associations and the importance of considering different added sugar sources when investigating health outcomes.

## Introduction

Cardiovascular disease (CVD) is responsible for one-third of all global deaths annually (1). Although the etiologies of CVDs vary, atherosclerosis is a common underlying mechanism, and there are many shared risk factors including hypertension, diabetes, hyperlipidemia, congenital heart defects and various lifestyle factors. In addition to the many shared risk factors, the presence of one CVD often increases the risk of developing another, which leads to high levels of comorbidity among individuals with CVD (2).

A poor diet is one of the main modifiable risk factors for CVD (2). Although added sugar consumption has been linked to various cardiometabolic risk markers (3, 4), the long-term effects and associations with incident CVD risk are not yet fully understood. Further, a majority of studies have primarily focused on the intake of sugar-sweetened beverages (SSBs) (3, 5), rather than the totality of added sugars. This is important because SSBs on average account for only 14% of added sugar intake in Sweden (6) and 23% in the US (7). The evidence regarding an association between SSB consumption and CVD risk is currently inconclusive. A systematic review by Keller et al. found that the evidence for an association between SSBs and vascular events was inconsistent, as two of three studies found an association with stroke and two of four studies found an association with coronary heart disease (8). Another systematic review reported inconclusive evidence regarding the association between sugar intake and SSBs and cardiovascular outcomes due to the low number of studies available (9).

It is hypothesized that SSB consumption could adversely impact health as a consequence of liquid calories causing insufficient satiety signaling and thereby promoting weight gain, a prominent risk factor for many CVDs (10). Another hypothesis is the distinct metabolism of fructose, one of the monosaccharides forming the commonly used added sugar sucrose. Some of the ingested fructose is metabolized into lipids in the liver, which is theorized to result in elevated triglyceride synthesis and increased hepatic lipid content, ultimately increasing the risk of CVD (11). Since different sources of added sugar may vary in composition, energy density, and absorption, it is important to distinguish between different sources of added sugar when studying their associations with health outcomes.

This study aims to investigate the associations between the intake of added sugar, as well as sugar-sweetened foods and beverages, and the risks of four different CVDs: stroke, coronary events, atrial fibrillation, and aortic stenosis.

## Materials and Methods

### Study Population

The study population is derived from the Malmö Diet and Cancer Study: a prospective population-based cohort study in southern Sweden. Recruitment was carried out through mailed invitation letters and distribution of invitations in public areas between 1991 and 1996. All men born between 1923 and 1945 and women born between 1923 and 1950 in Malmö were invited to participate, with the only exclusion criteria being insufficient Swedish language skills and mental incapacity. Of 68,905 individuals eligible for participation, 28,098 (40.8%) completed the baseline examinations. We excluded individuals with a history of aortic stenosis (*n* = 57), atrial fibrillation (*n* = 286), stroke (*n* = 300), coronary events (*n* = 544), and diabetes mellitus (*n* = 1,244) at baseline, resulting in a study sample of 25,877 individuals. A total of 139 individuals (81 women and 59 men) were lost to follow-up, with the primary reason being permanent emigration.

Ethical approval for the Malmö Diet and Cancer Study has been granted by the Regional Ethical Review Board in Lund, Sweden (LU/90-51), and written informed consent was obtained from all participants prior to participation.

### Data Collection

Baseline data collection was carried out using questionnaires covering medication, medical history, socioeconomic factors and current lifestyle, such as leisure-time physical activity (metabolic equivalent of task hours/week based on the duration and intensity of 17 different activities) (12), smoking status (never, current, or former smoker) and educational level according to Swedish educational degrees (<9, 9 years, upper secondary school, and university with or without a degree). Information about alcohol consumption was retrieved from the questionnaire as well as the 7-day food diary; zero-consumers indicated no consumption during the past year. The rest of the participants were divided into sex-specific quintiles based on their alcohol consumption reported in the 7-day food diary. Participants who reported drastic dietary changes prior to baseline examinations were identified through the self-administered questionnaires (13), and potential energy misreporters were identified using Black's revised Goldberg method (14) based on their estimated energy expenditure. Misreporting was defined as having an energy intake to basal metabolic rate ratio outside of the 95% confidence interval (CI) of the physical activity level (PAL). The procedure has been described in more detail previously (15).

Anthropometric measurements including weight, height, waist circumference, and body fat percentage were collected by registered nurses. Further, blood pressure was measured in all participants, and hypertension was determined according to the American Heart Association and the National Heart, Lung, and Blood Institute's definitions, which is a systolic blood pressure of ≥130 mmHg, diastolic blood pressure of ≥85 mm Hg, or use of antihypertensive drugs (16). Non-fasting blood samples were also collected, from which the concentrations of apolipoprotein B (ApoB) and apolipoprotein A-1 (ApoA-1) were determined using a Siemens BNII immunonephelometric analyzer during the year 2013 (Siemens, Newark, DE, USA) (17).

#### Dietary Assessment

Dietary data were collected using a modified diet history method that included a 7-day food diary covering cooked meals, cold beverages and dietary supplements as well as a 168-item diet history questionnaire covering the general meal pattern as well as the frequency and portion-size of non-cooked meals during the preceding 12 months. In addition, a 60-min (until September 1st, 1994) or 45-min (after September 1st, 1994) diet history interview was conducted to collect information about serving sizes and cooking methods of the foods recorded in the food diary. The participants' dietary intakes were estimated by adding up the reported intakes of the modified diet history method. The collected data were then converted into daily nutrient and energy intakes using the Malmö Diet and Cancer Study Food and Nutrient Database, originating from a database by the Swedish National Food Agency. A version of the modified diet history method was validated against an 18-day weighted food record, demonstrating a moderately strong correlation for sucrose, the most common added sugar in Sweden, with Pearson's correlation coefficients of 0.74 for women and 0.60 for men (18).

#### Added Sugar Variables

The participants' added sugar intakes were estimated based on the collected dietary data. The estimated sucrose and monosaccharide contents of the participants' reported intake of fruits and berries, vegetables, and juice were subtracted from their total intake of sucrose and monosaccharides to obtain an estimation of their added sugar intakes, which includes honey and syrup intake. This process has been explained in detail previously (19). Added sugar intake was then divided into six categories with focus on the current and suggested nutritional recommendations (20, 21) of 10 E% and 5 E%, respectively, as well as to allow extreme intakes to be studied. The categories were created according to percentage of non-alcoholic energy intake (E%) as follows: <5 E%, 5–7.5 E%, 7.5–10 E%, 10–15 E%, 15–20 E%, and >20 E% (19).

Treats included pastries, sweets, chocolate, and ice cream, while toppings included table sugar, syrups, honey and jams. Treats are generally considered more energy dense than toppings, as they tend to have higher fat contents, whereas toppings tend to have larger proportions of energy from sugar. As previous studies have shown that liquid sugar is metabolized differently and has different health outcomes from those associated with solid sugar (11) an SSB category was also created by combining the intake of carbonated and noncarbonated sweetened drinks and fruit drinks, but excluding the intake of pure fruit juice.

The consumed amounts of treats, toppings and SSBs were recoded from grams/day to servings/week based on average serving sizes according to the Swedish National Food Agency's food database and information from manufacturers (22). The consumption of treats was classified as ≤2, >2–5, >5–8, >8–14, and >14 servings/week; topping consumption was classified as ≤2, >2–7, >7–14, >14–28, and >28 servings/week; and SSB intake was classified as ≤1, >1–3, >3–5, >5–8, and >8 servings/week (19).

#### Endpoint Ascertainment

The participants were followed until diagnosis of the studied outcomes, death, emigration from Sweden or the end of the follow-up period (December 31st, 2016). Endpoints were ascertained using the Swedish National Inpatient Register and the Cause of Death Register, according to the International Classification of Diseases 9th revision (ICD-9) and the corresponding codes in the ICD-10. These registers include all residents of Sweden, and there was therefore no loss to follow-up during registry linkage.

The studied endpoints were stroke (ICD-9 codes 430, 431, 434, 436), coronary events (ICD-9 codes 410–414), atrial fibrillation (ICD-9 code 427 or code 4,273 in the Cause of Death Register), and aortic stenosis (ICD-9 code 424.1). Incident coronary events were defined as a diagnosis of myocardial infarction, other forms of ischemic heart disease or angina pectoris. Incident stroke was defined as a diagnosis of subarachnoid or intracerebral hemorrhage, occlusion of cerebral arteries or other acute cerebrovascular disease. Atrial fibrillation was defined as a diagnosis of either atrial fibrillation or flutter events. Aortic stenosis was defined as a diagnosis of aortic valve disorders. The Swedish National Inpatient Register has previously been shown to have high diagnostic validity, with positive predictive values over 90% for the studied outcomes (23, 24).

### Statistical Analyses

All analyses were conducted using IBM SPSS Statistics (version 24; IBM corporation, Armonk, NY, USA). *P* < 0.05 denoted statistical significance. Population characteristics were analyzed across the added sugar intake categories, using the chi-square test for categorical variables, and a univariate general linear model for continuous variables. Normally distributed continuous variables are expressed as means and standard deviations (SDs), while skewed continuous variables are expressed as medians and interquartile ranges (IQRs).

Cox hazards regression models with follow-up as time scale were used to study the associations between the intakes of added sugar, SSBs, treats and toppings and the risks of incident stroke, coronary events, atrial fibrillation, and aortic stenosis. The results are presented as hazard ratios (HRs) with 95% CI. The lowest intake category was generally used as reference, but wherever a U-shaped trend was observed, the intake category with the lowest risk was used as reference. The associations between the studied exposures and outcomes were investigated using four different models. The basic model was adjusted for age (years), sex, season of dietary assessment (spring, summer, autumn, winter), diet assessment method (45 or 60-min diet history interview), and total energy intake (kilocalories/day). The second model was further adjusted for the following lifestyle factors as categorical variables: smoking status, educational level, leisure-time physical activity, and alcohol consumption. The main model further included additional adjustment for body mass index (BMI) (kg/m^2^) categories and dietary factors, including intakes of processed meat (g/day), coffee (g/day), saturated fatty acids (E%), and fiber density (g/1,000 kilocalories). Adjustment for BMI separately without the dietary covariates was also tested, as it was suspected to be a particularly prominent confounder. The final model was further adjusted for potential mediators including the ApoB/ApoA-1 ratio, hypertension, and the use of lipid-lowering medications.

The proportional hazards assumptions were tested by plotting the partial residual plots for each variable against time using a scatterplot to see whether the hazards were proportional over time. To attain proportionality, all models were stratified by sex, as it was the most inconsistent covariate over time. A sensitivity analysis was conducted using the main model by excluding potential energy misreporters and individuals who had reported drastic diet changes prior to baseline assessments. In order to account for comorbidities, an additional sensitivity analysis was conducted by studying solely the first reported diagnosis for each participant. In this analysis, subjects who had experienced an incident event of another CVD prior to diagnosis of the specific outcome of interest in the analysis were excluded. In addition, participants with a prior incidence of diabetes mellitus (ICD-9 codes 150.0–150.9 or ICD-10 codes E10–E14) were also excluded in the comorbidity sensitivity analysis. The two sensitivity analyses were conducted both separately and combined.

## Results

### Population Characteristics

The study population consisted of 25,877 individuals aged 45–74 years (mean age of 57.8 years, 62.4% female). The mean added sugar intake was 10.1 E%, and the mean BMI was 25.6 kg/m^2^. During a mean follow-up of 19.5 years there were 2,580 stroke cases, 2,840 coronary events, 4,241 atrial fibrillation cases and 669 aortic stenosis cases. Individuals with high added sugar intake were more frequently male, older, and with lower BMI than individuals with low added sugar intake. Lower added sugar consumers tended to be overrepresented when it came to potential energy misreporting (primarily underreporting) and prior drastic diet changes, while they were generally more physically active and had a higher education level than higher added sugar consumers (Supplementary Table 1).

### Added Sugar and CVD Risk

In the main model, no linear associations were found between added sugar intake and the studied outcomes. However, a U-shaped trend was observed for added sugar intake and risk of incident stroke; consumers in the 7.5–10 E% group had the lowest risk, while increased risks were observed among the lowest (HR: 1.14; 95% CI: 0.97–1.34) and highest (HR: 1.31; 95% CI: 1.03–1.66) intake groups. For coronary events, an increased risk was observed for added sugar intakes above 20 E% compared to the lowest intake group (HR: 1.39; 95% CI: 1.09–1.78) (Table 1).

**Table 1:** Associations between intake of added sugar and risk of incident stroke, coronary events, atrial fibrillation and aortic stenosis.

	**Added sugar intake categories**						
	** <5 E%**	**5–7.5 E%**	**7.5–10 E%**	**10–15 E%**	**15–20 E%**	**>20 E%**	
	**(*n* = 2,354)**	**(*n* = 5,027)**	**(*n* = 6,709)**	**(*n* = 8,735)**	**(*n* = 2,377)**	**(*n* = 675)**	***P*-trend**
**Stroke**							
Cases/person-years	220/45,382	459/99,027	665/132,501	896/16,9957	251/45,522	89/12,129	
Basic model	1	0.84 (0.71–0.99)	0.84 (0.72–0.98)	0.85 (0.73–0.98)	0.88 (0.73–1.06)	1.28 (1.00–1.64)	0.62
Main model	1	0.87 (0.74–1.03)	0.88 (0.76–1.04)	0.87 (0.75–1.02)	0.89 (0.73–1.08)	1.16 (0.89–1.51)	0.39
Basic model^a^	1.19 (1.02–1.39)	1.00 (0.89–1.12)	1	1.00 (0.91–1.11)	1.05 (0.91–1.21)	1.52 (1.22–1.90)	–
Main model^a^	1.13 (0.97–1.33)	0.99 (0.87–1.11)	1	0.99 (0.89–1.10)	1.00 (0.86–1.17)	1.31 (1.03–1.66)	–
**Coronary events**							
Cases/person-years	216/45,758	526/98,903	712/132,271	1,000/169,782	271/45,356	115/12,192	
Basic model	1	1.01 (0.86–1.19)	0.96 (0.82–1.11)	1.01 (0.87–1.17)	1.01 (0.84–1.21)	1.73 (1.37–2.17)	0.02
Main model	1	1.02 (0.87–1.20)	0.99 (0.84–1.15)	1.00 (0.86–1.17)	0.92 (0.76–1.11)	1.39 (1.09–1.78)	0.47
**Atrial fibrillation**							
Cases/person-years	365/44,955	795/97,087	1,140/129,803	1,434/167,028	403/44,768	104/12,168	
Basic model	1	0.88 (0.78–1.00)	0.87 (0.77–0.98)	0.81 (0.72–0.91)	0.85 (0.74–0.99)	0.89 (0.71–1.10)	0.09
Main model	1	0.90 (0.80–1.03)	0.90 (0.80–1.02)	0.85 (0.75–0.96)	0.90 (0.78–1.05)	0.91 (0.72–1.15)	0.40
**Aortic stenosis**							
Cases/person-years	59/46,590	126/101,045	161/135,715	250/173,9587	53/46,720	20/12,632	
Basic model	1	0.85 (0.63–1.16)	0.74 (0.55–1.00)	0.86 (0.65–1.15)	0.68 (0.47–0.99)	1.05 (0.63–1.75)	0.46
Main model	1	0.86 (0.63–1.17)	0.76 (0.56–1.03)	0.89 (0.66–1.20)	0.69 (0.47–1.02)	0.97 (0.57–1.66)	0.84

a *Analysis of added sugar was carried out twice using different reference categories for stroke (<5 and 7.5–10 E%) due to the U-shaped trend. E%, Energy percentage*.

*The associations were determined using multivariable Cox proportional hazards regression model and are expressed as hazard ratio with a 95% confidence interval and P-value for the linear trend. The basic model was adjusted for age, sex, season of dietary assessment, diet method, and energy intake. The main model was adjusted for age, sex, season of dietary assessment, diet method, energy intake, smoking status, educational level, leisure-time physical activity, alcohol consumption, BMI, and dietary habits including intake of processed meat, coffee, saturated fatty acids, and fiber density*.

The lowest added sugar intake group had the highest risk of atrial fibrillation, with the lowest risk found among intakes of 10–15 E% (HR: 0.85; 95% CI: 0.75–0.96). Similarly, a borderline significant decreased risk of incident aortic stenosis was observed among intakes of 7.5–10 E% (HR: 0.75; 95% CI: 0.56–1.03) and 15–20 E% (HR: 0.69; 95% CI: 0.47–1.02) compared to the lowest intake group (Table 1). None of the associations were changed when the main model was further adjusted for the potential mediators ApoB/ApoA-1 ratio, hypertension, and the use of lipid-lowering medications (Supplementary Tables 2–5).

### Sugar-Sweetened Foods and Beverages and CVD Risk

**Figure 1:**
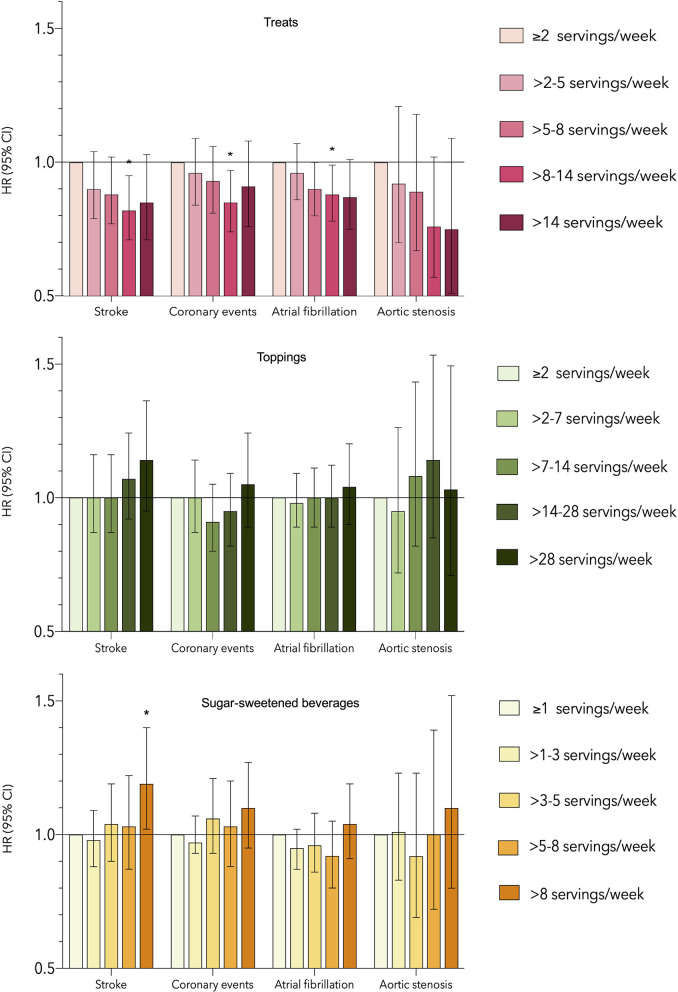
Associations between intake of treats, toppings, sugar-sweetened beverages, and risk of incident stroke, coronary events, atrial fibrillation and aortic stenosis using the main model. *Statistically significant association. HR, Hazard ratio; CI, Confidence interval.

The lowest intake group of treats (≤2 servings/week) was found to have the highest risks of stroke, coronary events, atrial fibrillation, and aortic stenosis. No associations were found between intake of toppings and any of the studied outcomes (Figure 1).

For SSBs, an increased risk of stroke was observed in the highest intake group (>8 servings/week) compared to the lowest intake group (<1 serving/week) (HR: 1.19; 95% CI: 1.01–1.40). No associations were found between the consumption of SSBs and incident coronary events, atrial fibrillation, or aortic stenosis (Figure 1).

### Sensitivity Analyses

When excluding potential energy misreporters and diet changers, the association between added sugar intake and stroke was strengthened, as an increased risk was observed in the highest intake group compared to the lowest intake group (HR:1.57; 95% CI: 1.12–2.19) (Table 2), as well as a positive linear trend (*P*-trend: 0.04). When further excluding incidence of the other diagnoses prior to diagnosis of each studied outcome, the association with stroke was additionally strengthened (HR: 1.70; 95% CI: 1.15–2.51; Table 3). Similarly, the association between added sugar and aortic stenosis was strengthened in the combined sensitivity analysis, in which a decreased risk was observed for intakes between 15 and 20 E% compared to the lowest intake (HR: 0.50; 95% CI: 0.26–0.97), and a borderline significant negative trend was found (*P*-trend: 0.07; Table 3). The association between added sugar intake and coronary events was attenuated when excluding potential energy misreporters and diet changers (Table 2).

**Table 2:** Sensitivity analysis excluding energy misreporters and participants who had made drastic dietary changes prior to baseline examinations, after which 16,781 participants remained.

		**Stroke**		**Coronary events**		**Atrial fibrillation**		**Aortic stenosis**	
**Intake**	***n***	**Cases/PY**	**HR (95% CI)**	**Cases/PY**	**HR (95% CI)**	**Cases/PY**	**HR (95% CI)**	**Cases/PY**	**HR (95% CI)**
**Added sugar, E%**									
<5	1,246	107/23,760	1	115/23,899	1	190/23,488	1	33/24,293	1
5–7.5	3,092	293/60,381	1.01 (0.81–1.27)	336/60,330	1.07 (0.87–1.33)	473/59,322	0.88 (0.75–1.05)	74/61,662	0.77 (0.51–1.17)
7.5–10	4,455	438/87,454	0.97 (0.78–1.21)	469/87,325	0.98 (0.80–1.20)	759/85,698	0.91 (0.77–1.07)	106/89,656	0.70 (0.47–1.04)
10–15	5,978	604/116,375	0.95 (0.76–1.18)	674/116,298	0.95 (0.78–1.17)	978/114,228	0.84 (0.71–0.99)	166/119,044	0.79 (0.53–1.17)
15–20	1,597	182/30,403	1.09 (0.84–1.41)	185/30,346	0.91 (0.71–1.16)	288/29,883	0.97 (0.80–1.18)	29/31,331	0.53 (0.31–0.89)
>20	413	65/7,417	1.57 (1.12–2.19)	67/7,460	1.28 (0.94–1.76)	63/7,535	0.86 (0.64–1.16)	11/7,787	0.77 (0.37–1.58)
*P*-trend			0.04		0.66		0.87		0.20
**Treats, servings/week**									
≤2	1,496	158/27,352	1	180/27,502	1	226/27,180	1	35/28,175	1
>2–5	4,236	408/82,251	0.85 (0.71–1.02)	453/82,303	0.92 (0.77–1.09)	667/80,850	0.97 (0.83–1.13)	107/84,070	0.96 (0.65–1.42)
>5–8	4,590	452/89,914	0.82 (0.68–0.99)	496/89,827	0.91 (0.76–1.09)	728/88,482	0.92 (0.79–1.07)	120/92,039	0.94 (0.63–1.38)
>8–14	4,869	505/95,240	0.80 (0.66–0.96)	526/95,233	0.82 (0.68–0.97)	846/93,307	0.92 (0.79–1.07)	119/97,639	0.77 (0.52–1.15)
>14	1,590	166/31,032	0.80 (0.63–1.01)	191/30,793	0.85 (0.68–1.06)	284/30,333	0.93 (0.77–1.12)	38/31,847	0.74 (0.45–1.21)
*P*-trend			0.12		0.01		0.61		0.08
**Toppings, servings/week**									
≤2	1,783	146/35,137	1	174/35,013	1	252/34,585	1	36/35,799	1
>2–7	4,237	381/83,694	0.99 (0.82–1.20)	386/83,971	0.91 (0.76–1.09)	622/82,515	0.96 (0.83–1.12)	90/85,626	0.97 (0.65–1.44)
>7–14	4,699	448/91,414	0.98 (0.81–1.18)	462/91,538	0.86 (0.72–1.03)	782/89,599	1.00 (0.87–1.16)	130/93,327	1.22 (0.83–1.79)
>14–28	4,216	476/81,319	1.06 (0.87–1.29)	526/81,030	0.90 (0.75–1.098)	763/79,466	0.98 (0.84–1.14)	119/83,542	1.17 (0.78–1.74)
>28	1,846	238/34,225	1.30 (1.04–1.63)	298/34,105	1.03 (0.84–1.27)	332/33,988	1.02 (0.85–1.22)	44/35,477	1.09 (0.68–1.77)
*P*-trend			<0.01		0.34		0.82		0.76
**Sugar-sweetened beverages, servings/week**									
≤1	9,609	968/185,693	1	1,041/185,809	1	1,608/182,216	1	243/190,184	1
>1–3	3,595	332/71,326	0.95 (0.84–1.08)	370/71,146	0.98 (0.87–1.11)	555/70,123	0.94 (0.86–1.04)	86/72,854	0.97 (0.76–1.25)
>3–5	1,524	146/29,797	0.95 (0.80–1.14)	170/29,672	0.99 (0.84–1.17)	246/29,191	0.98 (0.85–1.12)	40/30,446	1.02 (0.72–1.44)
>5–8	1,053	115/20,430	1.07 (0.87–1.30)	130/20,466	1.04 (0.86–1.25)	171/20,260	0.97 (0.82–1.13)	26/21,096	1.00 (0.66–1.50)
>8	1,000	128/18,542	1.24 (1.02–1.51)	135/18,565	1.03 (0.86–1.25)	171/18,362	1.01 (0.86–1.20)	24/19,191	0.94 (0.61–1.47)
*P*-trend			0.02		0.72		0.91		0.75

*E%, Energy percentage. PY, Person-years. HR, Hazard ratio. The associations were determined using multivariable Cox proportional hazards regression model and are expressed as HR with a 95% confidence interval and P-value for the linear trend. All analyses were carried out with adjustment according to the main model: age, sex, season of dietary assessment, diet method, energy intake, smoking status, educational level, leisure-time physical activity, alcohol consumption, body mass index, and dietary habits including intake of processed meat, coffee, saturated fatty acids, and fiber density*.

**Table 3:** Sensitivity analysis excluding diet changers, energy misreporters and individuals with prior incidence of other diagnoses (aortic stenosis, atrial fibrillation, stroke, coronary events or diabetes) for each particular outcome.

	**Stroke**		**Coronary events**		**Atrial fibrillation**		**Aortic stenosis**	
**Intake**	**n/Cases/PY**	**HR (95% CI)**	**n/Cases/PY**	**HR (95% CI)**	**n/Cases/PY**	**HR (95% CI)**	**n/Cases/PY**	**HR (95% CI)**
**Added sugar, E%**								
<5	897/79/16,723	1	867/77/16,374	1	955/136/17,862	1	840/22/16,183	1
5–7.5	2,158/204/41,212	0.98 (0.75–1.28)	2,138/242/41,078	1.13 (0.87–1.46)	2,297/343/43,653	0.94 (0.77–1.15)	2,009/54/39,546	0.88 (0.53–1.45)
7.5–10	3,091/392/59,891	0.94 (0.73–1.21)	3,049/326/58,896	1.02 (0.79–1.31)	3,349/561/63,995	0.98 (0.81–1.19)	2,858/69/57,113	0.69 (0.42–1.13)
10–15	4,174/421/80,383	0.90 (0.69–1.16)	4,137/479/79,858	0.96 (0.75–1.24)	4,452/697/84,944	0.86 (0.71–1.04)	3,854/101/76,795	0.70 (0.43–1.14)
15–20	1,103/132/20,934	1.08 (0.80–1.46)	1,086/138/20,370	0.99 (0.74–1.34)	1,181/209/21,924	1.02 (0.81–1.29)	990/18/19,388	0.50 (0.26–0.97)
>20	288/49/5,012	1.70 (1.15–2.51)	276/43/4,863	1.33 (0.89–1.98)	279/40/5,047	0.95 (0.66–1.38)	245/6/4,561	0.80 (0.31–2.07)
*P*-trend		0.05		0.78		0.73		0.07
**Treats, servings/week**								
≤2	1,064/112/18,968	1	1,035/126/18,619	1	1,105/152/19,854	1	978/26/18,144	1
>2–5	2,990/293/57,219	0.94 (0.75–1.17)	2,945/325/56,720	1.02 (0.82–1.25)	3,175/474/60,401	1.03 (0.86–1.24)	2,767/69/54,691	0.86 (0.54–1.37)
>5–8	3,258/317/63,057	0.88 (0.71–1.10)	3,220/352/62,303	0.97 (0.78–1.20)	3,473/533/66,584	0.99 (0.83–1.19)	3,016/73/60,236	0.78 (0.49–1.24)
>8–14	3,348/351/64,483	0.86 (0.68–1.07)	3,309/367/63,910	0.86 (0.69–1.06)	3,613/616/68,899	1.00 (0.83–1.21)	3,076/80/61,429	0.70 (0.44–1.13)
>14	1,051/114/20,127	0.88 (0.66–1.17)	1,044/135/19,887	0.93 (0.72–1.21)	1,147/211/21,688	1.09 (0.87–1.37)	959/22/19,087	0.62 (0.34–1.15)
P-trend		0.47		0.05		0.55		0.14
**Toppings, servings/week**								
≤2	1,289/92/25,032	1	1,290/125/25,126	1	1,378/180/26,594	1	1,221/23/24,338	1
>2–7	3,008/272/58,634	1.14 (0.90–1.45)	2,940/255/57,855	0.86 (0.69–1.07)	3,199/461/61,920	1.02 (0.86–1.21)	2,800/62/56,270	1.09 (0.67–1.79)
>7–14	3,323/326/63,710	1.12 (0.89–1.43)	3,259/339/62,762	0.88 (0.71–1.09)	3,581/585/68,068	1.05 (0.88–1.24)	3,078/87/60,949	1.25 (0.77–2.03)
>14–28	2,838/324/53,901	1.16 (0.91–1.49)	2,817/377/53,243	0.90 (0.73–1.12)	3,059/545/57,274	1.01 (0.84–1.21)	2,587/70/50,944	1.12 (0.67–1.85)
>28	1,253/173/22,578	1.39 (1.05–1.84)	1,247/209/22,452	0.94 (0.74–1.21)	1,296/215/23,570	0.98 (0.79–1.22)	1,110/28/21,084	1.11 (0.60–2.04)
*P*-trend		0.04		0.96		0.51		0.40
**SSBs, servings/week**								
≤1	6,751/671/128,328	1	6,654/734/126,912	1	7,270/1,190/136,921	1	6,233/153/122,511	1
>1–3	2,539/241/49,787	0.99 (0.85–1.14)	2,509/267/49,212	0.99 (0.86–1.14)	2,690/390/52,510	0.90 (0.80–1.01)	2,361/61/47,824	1.09 (0.80–1.47)
>3–5	1,048/105/20,183	1.03 (0.83–1.27)	1,041/123/20,063	1.04 (0.86–1.27)	1,118/175/21,189	0.98 (0.83–1.15)	969/27/19,276	1.13 (0.74–1.73)
>5–8	711/82/13,695	1.10 (0.87–1.39)	696/87/13,427	0.96 (0.77–1.21)	744/115/14,316	0.89 (0.73–1.08)	645/16/12,932	1.02 (0.60–1.72)
>8	662/88/11,862	1.30 (1.03–1.65)	653/94/11,824	1.08 (0.86–1.35)	691/116/12,491	1.01 (0.83–1.23)	588/13/11,041	0.98 (0.54–1.76)
*P*-trend		<0.01		0.92		0.90		0.72

*E%, Energy percentage. PY, Person-years. HR, Hazard ratio. SSBs, Sugar-sweetened beverages. The associations were determined using multivariable Cox proportional hazards regression model and are expressed as HR with a 95% confidence interval and P-value for the linear trend. All analyses were carried out with adjustment for age, sex, season of dietary assessment, diet method, energy intake, smoking status, educational level, leisure-time physical activity, alcohol consumption, body mass index, and dietary habits including intake of processed meat, coffee, saturated fatty acids, and fiber density*.

For treats, a majority of the associations were attenuated in the combined sensitivity analysis, though a tendency of the highest risk being found in the lowest intake group remained for stroke, coronary events, and aortic stenosis (Table 3). The association between topping intake and stroke was strengthened in the combined sensitivity analysis, in which an increased risk of stroke was observed in the highest intake group (HR: 1.39; 95% CI: 1.05–1.84). Similarly, the association for SSB intake was strengthened, as a positive trend (*P*-trend: <0.01) as well as an increased risk of stroke in the highest intake group (HR: 1.30; 95% CI: 1.03–1.65) was observed (Table 3).

## Discussion

We found that high added sugar intakes (>20 E%) were associated with increased risks of incident stroke and coronary events. A high intake of SSBs (>8 servings/week) could potentially explain part of the association with stroke. Contrary to our hypothesis, the lowest added sugar intake group (<5 E%) was indicated to have the highest risk of atrial fibrillation and aortic stenosis, and consumption of treats was negatively associated with risks of stroke, coronary events and atrial fibrillation.

Previous studies that have investigated the association between added sugar and incident CVD risk are lacking; however, there are a few studies that have examined the association with CVD mortality. Results from the NIH-AARP Diet and Health Study did not find an association between added sugar intake and risk of CVD mortality (25), although a tendency of a U-shaped association was observed. A U-shaped association has previously also been reported between added sugar intake and CVD mortality in the Malmö Diet and Cancer Study, with the highest risks being observed at ≥20 E% and second highest at <5 E (19). In a prospective cohort study of 11,733 US adults, added sugar was found to be linearly associated with CVD mortality when studying intakes between <10 and >25 E%, as intakes of >25 E% were associated with a 2.75-fold higher CVD mortality risk compared with <10 E% (5). The discrepancies between the study results could be explained by different consumption patterns, or that intakes below 5 E% were not studied separately in the latter study.

We observed a 19% increased risk of stroke in the highest intake group (>8 servings/week) of SSBs compared to the lowest intake group, while no associations were found for the other outcomes. The results from a Japanese prospective cohort study indicated that SSB consumption was associated with increased ischemic stroke risk, particularly among females, while no association was found with overall stroke or coronary events (26). It is therefore possible that the associations found in our study would have been even stronger if ischemic stroke cases were analyzed separately, though this hypothesis has yet to be tested. In contrast, the Male Health Professional Follow-up Study indicated a significant 20% increased risk of incident coronary heart disease among the highest quartile of SSB consumers compared to the lowest quartile, while no association with incident stroke was reported (27). The Nurses' Health Study, however, found associations between SSBs and both coronary heart disease and stroke (28, 29). Our study is, to the best of our knowledge, the first to investigate the association between the intake of SSBs and atrial fibrillation and aortic stenosis. As no differences were found when further adjusting the main model for the ApoB/ApoA-1 ratio, hypertension and use of lipid-lowering medication in our study, they are likely not strong mediating factors of the observed associations with added sugar, though a mediation analysis is required to confirm this. Previous results from the Malmö Diet and Cancer Study have shown a weak association between intake of sugar-sweetened foods and drinks and ApoB/ApoA (17). SSB consumption has been shown to adversely affect fasting blood glucose levels, inflammatory markers, and various blood lipids (30). Results from the Malmö Diet and Cancer Study have previously indicated an association between SSBs and circulating triglycerides (19); thus, it is possible that increased triglycerides could partly mediate the observed association between the intake of SSBs and the risk of incident stroke. Further, higher micronutrient dilution has been observed with very high free sugar intakes (>25 E%), as well as with very low intakes (<5 E%) (31), which could help explain the increased risks observed at both ends of the added sugar intake spectra the main results of our study. Significant inverse linear associations between added sugar and micronutrient intake have however been reported in the Malmö Diet and Cancer study (32), which is consistent with the results of the sensitivity analyses for stroke in this study. In our study, exclusion of participants who had reported prior drastic diet changes, and potential energy misreporters, resulted in an attenuation of the increased risks of stroke found at added sugar <5 E%. This indicates the role of dietary measurement error for the observed increased risks in the lowest intake category prior to sensitivity analyses.

The sensitivity analyses strengthened certain associations while attenuating others, ultimately emphasizing that dietary risk factors may vary between CVDs. In the sensitivity analysis where solely the first reported diagnosis of the included outcomes for each participant was studied, the association between stroke and added sugar was slightly strengthened, and the negative association between aortic stenosis and added sugar was strengthened. Thus, it is possible that not taking comorbidities into account could have steered the associations toward the null. This tendency might be due to the different etiologies of the studied diseases, highlighting the importance of taking comorbidity into consideration. For example, aortic stenosis has previously not been associated with dietary factors otherwise strongly associated with many cardiovascular diseases, such as dietary fiber intake or dietary patterns recommended for CVD prevention (33).

Diets commonly recommended to decrease the risk of CVD include the Mediterranean diet and The Dietary Approaches to Stop Hypertension (DASH) (34, 35). Both of the mentioned diets emphasize intake of unsaturated fats, lean meats and high-quality carbohydrates such as fruits and vegetables combined with limited intake of saturated fat, cholesterol and salt. Only the DASH diet explicitly recommends restricting added sugar intake, though generally, Mediterranean style diets tend to be low in added sugar as well (34, 35). As the associations between added sugar intake and CVD incidence are not yet fully known, we believe that the results of this study provide an important contribution to the future development of dietary guidelines for CVD prevention.

A major strength of this study was the large sample size, which allowed for rigorous sensitivity analyses. However, as the number of aortic stenosis cases was very low in the highest intake category, and especially in sensitivity analyses, an even larger study sample would have been beneficial for studying this outcome. Additional strengths of the Malmö Diet and Cancer Study include the comprehensive dietary assessment, as well as the ability to exclude diet changers and potential energy misreporters. Although the added sugar intakes in this study are based on estimations, the estimated intakes correspond well with those reported in national health surveys (6).

To isolate the studied diet-outcome associations, we adjusted for many confounders using a pre-specified model based on existing literature, though some residual confounding may exist. For example, reliable data on sodium intake, which national surveys have reported to exceed the recommended intakes (6, 36), and trans-fatty acid (TFA) intake were not available from the Malmö Diet and Cancer Study. Thus, they were not adjusted for despite them being established risk factors of CVD (1). Although the national average intake of TFA is ~0.5 E% (6), which is below the Food and Agriculture Organization of the United Nations recommended upper limit of 1 E% (37), it cannot be ruled out that the participants' intakes of trans-fatty acid intake or sodium have affected the results. Due to the nature of observational studies regarding risk of confounding, randomized controlled trials are ultimately required to be able to draw any conclusions about causality.

Baseline data were collected between 1991 and 1996, and lifestyle and dietary patterns may therefore differ from those of today's population. For example, the consumption of chocolate and confections has increased drastically between 1990 and 2017, and the consumption of SSBs has more than doubled during the same time period (38). Additionally, the consumption patterns of added sugar and sugar-sweetened foods and beverages may also differ between countries and age groups, further affecting the generalizability of the study results.

Dietary recommendations regarding added sugar intake vary globally, but the Nordic Nutrition Recommendations state that added sugar intake should not exceed 10 E%, with the basis mainly being prevention of caries, overweight, secondary diseases and micronutrient dilution (20, 32). The World Health Organization has a similar recommendation for free sugars, recommending it should be kept below 10 E%, and also suggest further reducing the intake to below 5 E% (21). According to a national survey from year 2010 to 2011, the average intake of added sugar in Sweden was estimated to be 9.6 E%, with ~40% of the Swedish population failing to achieve the national recommendation (6). The findings of this study do not support a reduction of the upper recommended limit of 10 E% to 5 E%, but a general reduction of added sugar intake and SSB consumption in the population could be beneficial for prevention of stroke and coronary events.

## Conclusion

The results of this study indicate that the associations vary, both between different CVDs and different sources of added sugar. Added sugar intakes of >20 E% were associated with increased risks of incident stroke and coronary events, while the lowest intake category of added sugar was indicated to have the highest risk of atrial fibrillation and aortic stenosis. High intakes of SSBs (>8 servings/week) were associated with increased stroke risk, while consumption of treats was negatively associated with risks of stroke, coronary events and atrial fibrillation. The findings indicate that a general reduction of added sugar and SSB consumption in the population could be beneficial for prevention of stroke and coronary events.

## Data Availability Statement

The datasets presented in this article are not readily available because of ethical and legal restrictions. Requests to access the datasets should be directed to the Chair of the Steering Committee for the Malmö cohorts, see instructions at https://www.malmo-kohorter.lu.se/english.

## Ethics Statement

The studies involving human participants were reviewed and approved by Regional Ethical Review Board in Lund, Sweden (LU/90-51). The patients/participants provided their written informed consent to participate in this study.

## Author Contributions

The study was devised by ES, and the analysis plan was constructed by SJ, ES, and SR. SJ conducted the analyses and drafted the manuscript, which was then critically revised by ES, SR, EG-P, and LJ. All authors helped in interpretation of the results, gave final approval, and agree to be accountable for all aspects of work ensuring integrity and accuracy.

## Conflict of Interest

The authors declare that the research was conducted in the absence of any commercial or financial relationships that could be construed as a potential conflict of interest.

## Abbreviations

ApoA-1: Apolipoprotein A-1
ApoB: Apolipoprotein B
BMI: Body Mass Index
CI: Confidence interval
CVD: Cardiovascular disease
E%: percentage of energy intake
HR: Hazard ratio
ICD-9: International Classification of Diseases 9
IQR: Interquartile range
SD: standard deviation
SSB: Sugar-sweetened beverage.
