# Supplementary material for: Associations Between Added Sugar Intake and Risk of Four Different Cardiovascular Diseases in a Swedish Population-Based Prospective Cohort Study
Source: Front Nutr. 2020 Dec 23;7:603653. doi: 10.3389/fnut.2020.603653 (PMC7786303; doi:10.3389/fnut.2020.603653)
Supplement: Supplementary file 1 [file Table_1.pdf]

**Supplemental table 1.** Baseline characteristics according to six categories of added sugar intake in energy percentage.

| | | Added sugar intake | | | | | |
|---|---|---|---|---|---|---|---|
| | | **<5 E%** | **5-7.5 E%** | **7.5-10 E%** | **10-15 E%** | **15-20 E%** | **≥20 E%** |
| | Median intake | 4.0% | 6.4% | 8.8% | 11.9% | 16.6% | 22.3% |
| Characteristics | N | 2,354 | 5,027 | 6,709 | 8,735 | 2,377 | 675 |
| **Mean (SD)** | | | | | | | |
| Age, years | | 56.0 (7.0) | 57.0 (7.4) | 58 (7.6) | 58.4 (7.7) | 58.4 (7.7) | 58.1 (7.3) |
| Energy intake, kcal/day | | 2026 (635.0) | 2177 (632.5) | 2277 (628.0) | 2356 (652.6) | 2422 (670.2) | 2468 (713.1) |
| BMI, kg/$m^2$ | | 26.2 (4.3) | 25.8 (4.0) | 25.6 (3.8) | 25.4 (3.8) | 25.1 (3.8) | 25.3 (4.3) |
| **N (%)** | | | | | | | |
| Female sex | | 1,508 (64.1) | 3,167 (63.0) | 4,217 (62.9) | 5,436 (62.2) | 1,418 (59.7) | 393 (58.2) |
| Use of lipid lowering drugs | | 52 (2.2) | 117 (2.3) | 139 (2.1) | 182 (2.1) | 53 (2.2) | 23 (3.4) |
| High LTPA (>50 MET-h/week) | | 4347 (14.9) | 711 (14.3) | 1,050 (15.8) | 1,488 (17.1) | 410 (17.3) | 109 (16.4) |
| High alcohol consumption (5th quintile) | | 617 (26.2) | 1,210 (24.1) | 1,314 (19.6) | 1,402 (16.1) | 317 (13.3) | 81 (12.0) |
| Current smokers | | 815 (34,6) | 1439 (28.6) | 1762 (26,3) | 2317 (26.5) | 749 (31.5) | 287 (28,5) |
| University degree | | 480 (20.4) | 826 (16.5) | 1,052 (15.7) | 1,102 (12.6) | 248 (10.5) | 73 (10.9) |
| Underreporters of energy intake | | 667 (28.3) | 995 (19.8) | 984 (14.7) | 992 (11.4) | 241 (10.1) | 64 (9.5) |
| Drastic diet changers | | 643 (27.4) | 1130 (22.5) | 1348 (20.1) | 1,745 (20.0) | 493 (25.9) | 174 (21.4) |
| **Median (IQR)** | | | | | | | |
| Treats, servings/week | | 2.38 (2.80) | 4.48 (3.85) | 6.36 (4.97) | 7.84 (6.18) | 8.87 (8.21) | 9.23 (10.0) |
| Toppings, servings/week | | 1.79 (3.50) | 5.04 (6.07) | 8.69 (9.21) | 14.1 (14.3) | 21.5 (24.0) | 28.2 (35.6) |
| SSB, servings/week | | 0.00 (0.00) | 0.00 (0.71) | 0.05 (1.47) | 1.18 (3.57) | 3.57 (7.30) | 7.68 (13.2) |

E%: Energy percentage. SD: Standard deviation. BMI: Body mass index. MET: Metabolic equivalent of task. LTPA: Leisure-time physical activity. IQR: Interquartile range. SSB: Sugar-sweetened beverages. The univariate general linear model and chi squared-test were used to study the baseline characteristics according to six categories of sugar intake. Continuous variables are presented as mean (SD) or median (IQR) and categorical variables are presented as mean with percentage (%).
