# Supplementary material for: Associations Between Added Sugar Intake and Risk of Four Different Cardiovascular Diseases in a Swedish Population-Based Prospective Cohort Study
Source: Front Nutr. 2020 Dec 23;7:603653. doi: 10.3389/fnut.2020.603653 (PMC7786303; doi:10.3389/fnut.2020.603653)
Supplement: Supplementary file 2 [file Table_2.pdf]

Supplemental table 2. Associations between intake of added sugar, treats, toppings and SSBs and risk of incident stroke for various covariate adjustment models. Analysis of added sugar was carried out twice using different reference categories (<5 E% and 7.5-10 E%).

**Stroke**<br>Intake	n/Cases/person-years	Basic model with lifestyle factors[1]<br>HR (95% CI)	Basic model with lifestyle factors and BMI[2]<br>HR (95% CI)	Main model with potential mediators[3]<br>HR (95% CI)
Added sugar, E%				
<5	2,354/220/45,382	1	1	1
5-7.5	5,027/459/99,027	0.86 (0.73-1.02)	0.88 (0.75-1.03)	0.88 (0.75-1.04)
7.5-10	6,709/665/132,501	0.87 (0.74-1.01)	0.90 (0.77-1.05)	0.89 (0.76-1.05)
10-15	8,735/896/16,9957	0.87 (0.75-1.01)	0.89 (0.77-1.04)	0.88 (0.75-1.03)
15-20	2,377/251/45,522	0.87 (0.74-1.08)	0.92 (0.76-1.10)	0.88 (0.72-1.07)
>20	675/89/12,129	1.24 (0.96-1.60)	1.22 (0.94-1.58)	1.13 (0.86-1.48)
P-trend		0.83	0.16	0.60
Added sugar, E%				
<5	2,354/220/45,382	1.13 (0.96-1.32)	1.12 (0.96-1.31)	1.13 (0.96-1.33)
5-7.5	5,027/459/99,027	0.98 (0.87-1.11)	0.98 (0.87-1.11)	0.99 (0.88-1.12)
7.5-10	6,709/665/132,501	1	1	1
10-15	8,735/896/16,9957	0.99 (0.89-1.09)	1.00 (0.90-1.10)	0.98 (0.88-1.08)
15-20	2,377/251/45,522	1.01 (0.87-1.17)	1.02 (0.88-1.18)	0.98 (0.84-1.14)
>20	675/89/12,129	1.35 (1.07-1.69)	1.36 (1.08-1.71)	1.26 (0.99-1.60)
Treats, servings/week				
≤2	2,931/293/54,977	1	1	1
>2-5	7,161/685/140,388	0.91 (0.79-1.05)	0.91 (0.79-1.05)	0.91 (0.79-1.05)
>5-8	6,775/672/133,224	0.89 (0.78-1.03)	0.90 (0.78-1.04)	0.88 (0.76-1.01)
>8-14	6,687/680/131,146	0.84 (0.72-0.97)	0.84 (0.73-0.97)	0.82 (0.71-0.95)
>14	2,323/250/44,781	0.88 (0.73-1.06)	0.88 (0.73-1.06)	0.85 (0.70-1.03)
P-trend		0.10	0.10	0.03
Toppings, servings/week				
≤2	3,419/280/67,882	1	1	1
>2-7	7,008/630/138,604	1.00 (0.87-1.15)	1.01 (0.87-1.16)	1.00 (0.86-1.16)
>7-14	6,909/675/135,107	0.99 (0.86-1.14)	1.01 (0.87-1.16)	1.00 (0.87-1.16)

>14-28	5,908/677/114,003	1.05 (0.90-1.21)	1.08 (0.93-1.25)	1.08 (0.93-1.26)
>28	2,633/318/48,921	1.12 (0.94-1.33)	1.16 (0.97-1.39)	1.13 (0.94-1.36)
P-trend		0.27	0.12	0.26
SSBs, servings/week				
≤1	15,188/1,507/295,939	1	1	1
>1-3	5,329/494/105,894	1.00 (0.90-1.10)	0.99 (0.89-1.09)	0.98 (0.89-1.09)
>3-5	2,246/230/43,889	1.06 (0.92-1.22)	1.05 (0.91-1.21)	1.02 (0.88-1.17)
>5-8	1,561/159/30,144	1.05 (0.89-1.24)	1.04 (0.88-1.23)	1.00 (0.84-1.18)
>8	1,553/190/28,652	1.24 (1.06-1.44)	1.22 (1.04-1.42)	1.17 (0.99-1.38)
P-trend		<0.01	<0.01	0.02

BMI: Body Mass Index. E%: Energy percentage. MET: Metabolic equivalent of task. HR: Hazard ratio. CI: Confidence interval. SSBs: Sugar-sweetened beverages.

[1] Adjusted for age, sex, season of dietary assessment, diet method, energy intake, smoking status, educational level, leisure-time physical activity and alcohol consumption.

[2] Adjusted for age, sex, season of dietary assessment, diet method, energy intake, smoking status, educational level, leisure-time physical activity, alcohol consumption and body mass index.

[3] Adjusted for age, sex, season of dietary assessment, diet method, energy intake, smoking status, educational level, leisure-time physical activity, alcohol consumption, body mass index and dietary habits including intake of processed meat, coffee, saturated fatty acids and fiber density, ApoB/ApoA-1, hypertension and lipid-lowering medication.

The associations were determined using multivariable Cox proportional hazards regression model and are expressed as HR with a 95% confidence interval and P-value for the linear trend.
