# Supplementary material for: Associations Between Added Sugar Intake and Risk of Four Different Cardiovascular Diseases in a Swedish Population-Based Prospective Cohort Study
Source: Front Nutr. 2020 Dec 23;7:603653. doi: 10.3389/fnut.2020.603653 (PMC7786303; doi:10.3389/fnut.2020.603653)
Supplement: Supplementary file 3 [file Table_3.pdf]

Supplemental table 3. Associations between intake of added sugar, treats, toppings and sugar sweetened beverages and risk of incident coronary events for various covariate adjustment models.

**Coronary events**<br>Intake	n/Cases/Person-years	Basic model with lifestyle factors[1]<br>HR (95% CI)	Basic model with lifestyle factors and BMI[2]<br>HR (95% CI)	Main model with potential mediators[3]<br>HR (95% CI)
Added sugar				
<5 E%	2,354/216/45,758	1	1	1
5-7.5 E%	5,027/526/98,903	1.04 (0.88-1.22)	1.04 (0.89-1.23)	1.03 (0.87-1.21)
7.5-10 E%	6,709/712/132,271	1.01 (0.86-1.18)	1.02 (0.88-1.21)	0.98 (0.84-1.15)
10-15 E%	8,735/1,000/169,782	1.03 (0.89-1.20)	1.06 (0.92-1.25)	1.00 (0.85-1.17)
15-20 E%	2,377/271/45,356	0.97 (0.80-1.16)	1.00 (0.83-1.20)	0.90 (0.74-1.09)
>20 E%	675/115/12,192	1.54 (1.22-1.94)	1.58 (1.26-1.99)	1.36 (1.06-1.74)
P-trend		0.18	0.02	0.64
Treats, servings/week				
≤2	2,931/338/55,191	1	1	1
>2-5	7,161/764/140,332	0.97 (0.85-1.10)	0.97 (0.85-1.10)	0.95 (0.83-1.08)
>5-8	6,775/724/133,183	0.94 (0.82-1.07)	0.95 (0.83-1.08)	0.91 (0.79-1.04)
>8-14	6,687/728/131,00	0.86 (0.75-0.99)	0.88 (0.76-1.00)	0.85 (0.74-0.97)
>14	2,323/286/44,553	0.95 (0.80-1.13)	0.95 (0.80-1.13)	0.88 (0.74-1.05)
P-trend		0.15	0.16	0.02
Toppings, servings/week				
≤2	3,419/319/67,838	1	1	1
>2-7	7,008/676/138,746	1.00 (0.87-1.14)	1.01 (0.88-1.16)	0.96 (0.84-1.11)
>7-14	6,909/692/135,383	0.92 (0.80-1.05)	0.94 (0.82-1.08)	0.91 (0.79-1.04)
>14-28	5,908/735/113,631	0.95 (0.83-1.09)	0.99 (0.86-1.14)	0.92 (0.80-1.06)
>28	2,633/418/48,663	1.06 (0.90-1.25)	1.12 (0.95-1.32)	1.01 (0.86-1.20)
P-trend		0.23	0.06	0.35
SSBs, servings/week				
≤1	15,188/1,634/295,913	1	1	1
>1-3	5,329/535/105,722	0.99 (0.90-1.09)	0.99 (0.89-1.09)	0.96 (0.87-1.06)
>3-5	2,246/267/43,789	1.10 (0.97-1.25)	1.09 (0.96-1.24)	1.02 (0.89-1.17)

>5-8	1,561/185/30,137	1.08 (0.93-1.26)	1.07 (0.92-1.25)	1.04 (0.89-1.21)
>8	1,553/219/28,700	1.20 (1.03-1.38)	1.17 (1.01-1.36)	1.09 (0.94-1.27)
P-trend		0.002	0.005	0.09

BMI: Body Mass Index. E%: Energy percentage. MET: Metabolic equivalent of task. HR: Hazard ratio. CI: Confidence interval. SSBs: Sugar-sweetened beverages.

[1] Adjusted for age, sex, season of dietary assessment, diet method, energy intake, smoking status, educational level, leisure-time physical activity and alcohol consumption.

[2] Adjusted for age, sex, season of dietary assessment, diet method, energy intake, smoking status, educational level, leisure-time physical activity, alcohol consumption and body mass index.

[3] Adjusted for age, sex, season of dietary assessment, diet method, energy intake, smoking status, educational level, leisure-time physical activity, alcohol consumption, body mass index and dietary habits including intake of processed meat, coffee, saturated fatty acids and fiber density, ApoB/ApoA-1, hypertension and lipid-lowering medication.

The associations were determined using multivariable Cox proportional hazards regression model and are expressed as HR with a 95% confidence interval and P-value for the linear trend.
