# Supplementary material for: Associations Between Added Sugar Intake and Risk of Four Different Cardiovascular Diseases in a Swedish Population-Based Prospective Cohort Study
Source: Front Nutr. 2020 Dec 23;7:603653. doi: 10.3389/fnut.2020.603653 (PMC7786303; doi:10.3389/fnut.2020.603653)
Supplement: Supplementary file 4 [file Table_4.pdf]

Supplemental table 4. Associations between intake of added sugar, treats, toppings and sugar sweetened beverages and risk of incident atrial fibrillation for various covariate adjustment models.

**Atrial fibrillation**<br>Intake	n/Cases/person-years	Basic model with lifestyle factors[1]<br>HR (95% CI)	Basic model with lifestyle factors and BMI[2]<br>HR (95% CI)	Main model with potential mediators[3]<br>HR (95% CI)
Added sugar, E%				
<5	2,354/365/44,955	1	1	1
5-7.5	5,027/795/97,087	0.89 (0.79-1.01)	0.91 (0.80-1.03)	0.93 (0.82-1.06)
7.5-10	6,709/1,140/129,803	0.87 (0.79-1.00)	0.91 (0.81-1.03)	0.92 (0.82-1.04)
10-15	8,735/1,434/167,028	0.83 (0.74-0.94)	0.86 (0.77-0.97)	0.88 (0.78-0.99)
15-20	2,377/403/44,768	0.88 (0.76-1.01)	0.92 (0.80-1.07)	0.93 (0.80-1.09)
>20	675/104/12,168	0.91 (0.73-1.14)	0.94 (0.75-1.17)	0.91 (0.72-1.15)
P-trend		0.20	0.59	0.53
Treats, servings/week				
≤2	2,931/459/54,387	1	1	1
>2-5	7,161/1,152/137,977	0.97 (0.86-1.08)	0.97 (0.87-1.08)	0.97 (0.86-1.08)
>5-8	6,775/1,083/131,129	0.89 (0.80-1.00)	0.91 (0.81-1.01)	0.89 (0.79-1.00)
>8-14	6,687/1,149/128,388	0.88 (0.79-0.99)	0.89 (0.80-1.00)	0.88 (0.78-0.99)
>14	2,323/398/43,930	0.89 (0.77-1.03)	0.89 (0.77-1.03)	0.87 (0.75-1.02)
P-trend		0.09	0.09	0.05
Toppings, servings/week				
≤2	3,419/489/66,757	1	1	1
>2-7	7,008/1,052/136,751	0.97 (0.87-1.08)	0.98 (0.88-1.09)	0.97 (0.87-1.09)
>7-14	6,909/1,151/132,485	0.97 (0.87-1.08)	1.00 (0.90-1.12)	1.00 (0.90-1.12)
>14-28	5,908/1.070/111,456	0.95 (0.85-1.06)	1.01 (0.90-1.13)	1.00 (0.89-1.13)
>28	2,633/479/48,361	0.97 (0.85-1.12)	1.05 (0.91-1.20)	1.05 (0.91-1.22)
P-trend		0.71	0.44	0.36
SSBs, servings/week				
≤1	15,188/2,543/290,321	1	1	1
>1-3	5,329/824/104,252	0.96 (0.89-1.04)	0.95 (0.88-1.03)	0.95 (0.88-1.03)
>3-5	2,246/363/43,154	0.99 (0.88-1.04)	0.97 (0.87-1.08)	0.98 (0.87-1.10)

>5-8	1,561/241/29,855	0.94 (0.82-1.08)	0.93 (0.81-1.06)	0.92 (0.80-1.05)
>8	1,553/270/28,228	1.08 (0.95-1.23)	1.05 (0.92-1.20)	1.04 (0.91-1.19)
P-trend		0.44	0.79	0.85

BMI: Body Mass Index. E%: Energy percentage. MET: Metabolic equivalent of task. HR: Hazard ratio. CI: Confidence interval. SSBs: Sugar-sweetened beverages.

[1] Adjusted for age, sex, season of dietary assessment, diet method, energy intake, smoking status, educational level, leisure-time physical activity and alcohol consumption.

[2] Adjusted for age, sex, season of dietary assessment, diet method, energy intake, smoking status, educational level, leisure-time physical activity, alcohol consumption and body mass index.

[3] Adjusted for age, sex, season of dietary assessment, diet method, energy intake, smoking status, educational level, leisure-time physical activity, alcohol consumption, body mass index and dietary habits including intake of processed meat, coffee, saturated fatty acids and fiber density, ApoB/ApoA-1, hypertension and lipid-lowering medication.

The associations were determined using multivariable Cox proportional hazards regression model and are expressed as HR with a 95% confidence interval and P-value for the linear trend.
