# Supplementary material for: Associations Between Added Sugar Intake and Risk of Four Different Cardiovascular Diseases in a Swedish Population-Based Prospective Cohort Study
Source: Front Nutr. 2020 Dec 23;7:603653. doi: 10.3389/fnut.2020.603653 (PMC7786303; doi:10.3389/fnut.2020.603653)
Supplement: Supplementary file 5 [file Table_5.pdf]

Supplemental table 5. Associations between intake of added sugar, treats, toppings and sugar sweetened beverages and risk of incident aortic stenosis for various covariate adjustment models.

**Aortic stenosis**<br>Intake	n/Cases/person-years	Basic model with lifestyle factors[1]<br>HR (95% CI)	Basic model with lifestyle factors and BMI[2]<br>HR (95% CI)	Main model with potential mediators[3]<br>HR (95% CI)
Added sugar, E%				
<5	2,354/59/46,590	1	1	1
5-7.5	5,027/126/101,045	0.84 (0.62-1.15)	0.86 (0.63-1.18)	0.87 (0.63-1.20)
7.5-10	6,709/161/135,715	0.74 (0.55-1.00)	0.77 (0.57-1.05)	0.78 (0.57-1.06)
10-15	8,735/250/173,9587	0.87 (0.65-1.16)	0.92 (0.69-1.23)	0.90 (0.66-1.22)
15-20	2,377/53/46,720	0.68 (0.47-1.00)	0.74 (0.50-1.07)	0.69 (0.46-1.02)
>20	675/20/12,632	1.05 (0.63-1.75)	1.09 (0.65-1.82)	0.89 (0.51-1.55)
P-trend		0.52	0.74	0.70
Treats, servings/week				
≤2	2,931/74/56,470	1	1	1
>2-5	7,161/183/143,543	0.92 (0.70-1.21)	0.93 (0.71-1.22)	0.90 (0.68-1.19)
>5-8	6,775/181/136,355	0.90 (0.68-1.19)	0.92 (0.69-1.21)	0.87 (0.66-1.16)
>8-14	6,687/171/134,249	0.77 (0.58-1.03)	0.79 (0.59-1.05)	0.76 (0.57-1.02)
>14	2,323/60/46,044	0.79 (0.55-1.15)	0.80 (0.55-1.15)	0.73 (0.49-1.06)
P-trend		0.12	0.13	0.05
Toppings, servings/week				
≤2	3,419/74/69,234	1	1	1
>2-7	7,008/156/141,771	0.93 (0.71-1.24)	0.95 (0.72-1.26)	0.90 (0.68-1.20)
>7-14	6,909/187/138,010	1.03 (0.78-1.36)	1.09 (0.82-1.43)	1.08 (0.82-1.43)
>14-28	5,908/182/117.102	1.05 (0.79-1.40)	1.14 (0.86-1.52)	1.14 (0.85-1.53)
>28	2,633/70/50,543	0.96 (0.67-1.37)	1.07 (0.74-1.53)	1.01 (0.70-1.46)
P-trend		0.87	0.62	0.83
SSBs, servings/week				
≤1	15,188/394/302,807	1	1	1
>1-3	5,329/135/108,253	1.04 (0.85-1.26)	1.02 (0.84-1.24)	1.02 (0.83-1.24)
>3-5	2,246/55/45,008	0.96 (0.72-1.28)	0.94 (0.70-1.25)	0.91 (0.68-1.22)

>5-8	1,561/40/31,046	1.04 (0.75-1.45)	1.01 (0.73-1.41)	1.01 (0.72-1.41)
>8	1,553/45/29,547	1.18 (0.86-1.62)	1.13 (0.83-1.56)	1.05 (0.75-1.47)
P-trend		0.57	0.79	0.92

BMI: Body Mass Index. E%: Energy percentage. MET: Metabolic equivalent of task. HR: Hazard ratio. CI: Confidence interval. SSBs: Sugar-sweetened beverages.

[1] Adjusted for age, sex, season of dietary assessment, diet method, energy intake, smoking status, educational level, leisure-time physical activity and alcohol consumption.

[2] Adjusted for age, sex, season of dietary assessment, diet method, energy intake, smoking status, educational level, leisure-time physical activity, alcohol consumption and body mass index.

[3] Adjusted for age, sex, season of dietary assessment, diet method, energy intake, smoking status, educational level, leisure-time physical activity, alcohol consumption, body mass index and dietary habits including intake of processed meat, coffee, saturated fatty acids and fiber density, ApoB/ApoA-1, hypertension and lipid-lowering medication.

The associations were determined using multivariable Cox proportional hazards regression model and are expressed as HR with a 95% confidence interval and P-value for the linear trend.
