# Supplementary material for: Associations Between Added Sugar Intake and Risk of Four Different Cardiovascular Diseases in a Swedish Population-Based Prospective Cohort Study
Source: Front Nutr. 2020 Dec 23;7:603653. doi: 10.3389/fnut.2020.603653 (PMC7786303; doi:10.3389/fnut.2020.603653)
Supplement: Supplementary file 6 [file Table_6.pdf]

Supplemental table 6. Sensitivity analysis solely studying the first reported diagnosis for each participant, excluding incidence of the other diagnoses, and diabetes mellitus, prior to diagnosis of each of the studied outcome.

	Stroke		Coronary events		Atrial fibrillation		Aortic stenosis	
Intake	n/Cases/PY	HR (95% CI)	n/Cases/PY	HR (95% CI)	n/Cases/PY	HR (95% CI)	n/Cases/PY	HR (95% CI)
Added sugar, E%								
<5	2,292/158/44,438	1.19 (0.98-1.43)	2,284/146/44,688	1	2,249/260/43,196	1	2,331/36/46,244	1
5-7.5	4,882/314/96,756	1.00 (0.87-1.15)	4,875/374/96,528	1.09 (0.90-1.33)	4,792/560/93,233	0.90 (0.77-1.04)	4,986/85/100,368	0.94 (0.63-2.40)
7.5-10	6,500/456/129,239	1.02 (0.90-1.15)	6,499/502/128,845	1.06 (0.87-1.28)	6381/812/124,527	0.90 (0.78-1.04)	6,651/103/134,686	0.77 (0.52-1.15)
10-15	8,455/616/165,858	1	8,433/698/165,236	1.05 (0.87-1.26)	8,309/1,008/16,0312	0.84 (0.72-0.97)	8,635/150/172,355	0.86 (0.58-1.26)
15-20	2,307/181/44,372	1.06 (0.90-1.26)	2,307/201/44,228	1.01 (0.80-1.26)	2,267/293/42,948	0.93 (0.77-1.11)	2,356/32/46,342	0.68 (0.41-1.13)
>20	650/64/11,714	1.38 (1.05-1.81)	633/73/11,600	1.37 (1.01-1.85)	640/69/11,606	0.88 (0.67-1.16)	667/12/12,484	0.99 (0.49-1.97)
P-trend		-		0.61		0.37		0.66
Treats, servings/week								
≤2	2,844/206/53,758	1	2,828/235/53,614	1	2,791/319/52,082	1	2,902/45/55,992	1
>2-5	6,955/479/137,175	0.91 (0.77-1.08)	6,937/540/136,831	1.01 (0.86-1.18)	6,815/806/132,336	0.95 (0.84-1.09)	7,093/115/142,400	0.94 (0.66-1.34)
>5-8	6,562/459/129,905	0.87 (0.73-1.03)	6,560/509/129,850	0.97 (0.82-1.13)	6,447/755/125,930	0.88 (0.77-1.00)	6,706/112/135,258	0.89 (0.62-1.27)
>8-14	6,479/472/127,939	0.83 (0.70-0.99)	6,471/512/127,570	0.89 (0.75-1.04)	6,362/824/123,115	0.90 (0.78-1.03)	6,627/111/133,196	0.80 (0.55-1.16)
>14	2,246/173/43,601	0.84 (0.67-1.06)	2,235/198/43,260	0.94 (0.76-1.16)	2,223/298/42,359	0.95 (0.79-1.13)	2,298/35/4,5633	0.73 (0.45-1.18)
P-trend		0.12		0.09		0.45		0.16
Toppings, servings/week								
≤2	3,319/180/66,328	1	3,319/219/66,283	1	3,272/342/64,382	1	3,385/40/68,685	1
>2-7	6,817/439/135,667	1.08 (0.91-1.29)	6,786/454/135,282	0.99 (0.84-1.17)	6,708/752/131,800	0.98 (0.86-1.12)	6,958/106/140,905	1.22 (0.84-1.76)
>7-14	6,705/471/131,948	1.09 (0.91-1.30)	6,711/494/132,230	0.96 (0.81-1.13)	6,603/845/127,577	1.03 (0.90-1.17)	6,843/121/136,909	1.30 (0.90-1.89)
>14-28	5,698/467/110,833	1.17 (0.97-1.41)	5,702/529/110,508	0.99 (0.84-1.17)	5,587/749/106,407	1.00 (0.87-1.14)	5,834/108/115,881	1.31 (0.88-1.94)
>28	2,547/232/47,601	1.28 (1.02-1.60)	2,513/298/46,823	1.07 (0.88-1.30)	2,468/314/45,656	1.00 (0.85-1.19)	2,606/43/50,100	1.25 (0.77-2.02)
	P-trend	0.11		0.32		0.99		0.80
SSBs, servings/week								
≤1	14,7161,035/288,745	1	14,704/1,150/288,292	1	14,480/1,835/279,047	1	15,043/249/300,474	1
>1-3	5,188/353/103,743	1.03 (0.91-1.16)	5,180/386/103,402	0.98 (0.87-1.10)	5,085/580/100,242	0.93 (0.85-1.02)	5,279/85/107,378	1.03 (0.80-1.33)
>3-5	2,180/164/42,821	1.09 (0.92-1.29)	2,164/185/42,487	1.04 (0.89-1.22)	2,125/242/41,193	0.90 (0.79-1.03)	2,228/37/44,706	1.01 (0.71-1.45)
>5-8	1,512110/29,433	1.06 (0.86-1.29)	1,503/127/29,290	0.99 (0.82-1.19)	1,482/162/28,565	0.87 (0.74-1.03)	1,545/24/30,751	1.01 (0.66-1.55)
>8	1,490/127/27,634	1.18 (0.97-1.44)	1,480/146/27,655	1.03 (0.86-1.24)	1,466/183/26,775	1.01 (0.86-1.19)	1,531/23/29,171	0.98 (0.62-1.52)

P-trend	0.01	0.72	0.66	0.77

E%: Energy percentage. PY: Person-years. HR: Hazard ratio. SSBs: Sugar-sweetened beverages.

The associations were determined using multivariable Cox proportional hazards regression model and are expressed as HR with a 95% confidence interval and P-value for the linear trend. All analyses were carried out with adjustment for age, sex, season of dietary assessment, diet method, energy intake, smoking status, educational level, leisure-time physical activity, alcohol consumption, body mass index and dietary habits including intake of processed meat, coffee, saturated fatty acids and fiber density.
